# Designing and developing a prescription digital therapeutic for at-home heart rate variability biofeedback to support and enhance patient outcomes in post-traumatic stress disorder treatment

**DOI:** 10.3389/fdgth.2025.1503361

**Published:** 2025-02-11

**Authors:** Rebecca Macy, Flavio Somanji, Oleksandr Sverdlov

**Affiliations:** ^1^ School of Arts and Sciences, Massachusetts College of Pharmacy and Health Sciences, Boston, MA, United States; ^2^ School of Healthcare Business and Technology, Massachusetts College of Pharmacy and Health Sciences, Boston, MA, United States; ^3^ Biomedical Research, Novartis, Cambridge, MA, United States; ^4^ Early Development Analytics, Novartis Pharmaceutical Corporation, East Hanover, NJ, United States

**Keywords:** post-traumatic stress disorder (PTSD), heart rate variability biofeedback (HRV-BFB), prescription digital therapeutic (PDT), digital therapeutic, psychological therapies

## Abstract

Post-traumatic stress disorder (PTSD) is a psychiatric condition producing considerable distress, dysfunction, and impairment in affected individuals. While various forms of psychotherapy are commonly utilized in PTSD treatment, the known neurological pathologies associated with PTSD are insufficiently addressed by these conventional approaches. Heart rate variability biofeedback (HRV-BFB) is a promising tool for correcting autonomic dysfunction in PTSD, with subsequent changes in clinically significant outcome measures. This paper outlines a systematic approach for the development, distribution, and implementation of a prescription at-home HRV-BFB digital therapeutic. We provide recommendations for evidence-generation strategies and propose appropriate regulatory pathways within existing frameworks. Widespread access to HRV-BFB could potentially reduce the distress, disability, and healthcare burden associated with PTSD. Promoting HRV-BFB as a primary intervention could also serve to reduce the stigma associated with “mental” illness and increase health literacy regarding the neuroimmune impacts of psychosocial factors. These processes might in turn improve treatment-seeking, adherence, and supported self-management of these conditions.

## 1 Introduction

Digital therapeutics (DTx) represent a broad class of digital health technologies that have a therapeutic effect on patients (1, 2). In contrast to general wellness technologies or diagnostic devices, many DTx products are used to manage or treat medical conditions. Therefore, similar to traditional drugs and biologics, candidate DTx products should undergo rigorous testing in clinical trials, in compliance with Good Clinical Practice (GCP), and undergo review and approval by health authorities (3).

There are numerous ways in which a particular DTx solution can be developed for a given indication. Two important considerations are the mechanism of action (“software that treats”) and the form of delivery. For instance, the mechanism of action can be a cognitive behavioral therapy (CBT) to improve sleep-related behaviors and thoughts to treat insomnia (4), or some motivational enhancement techniques to help individuals with substance use disorders (5). Alternatively, physiological interventions such as neurostimulation or biofeedback, or closed-loop delivery systems that allow health monitoring and dose adjustments, such as artificial pancreas (6), can be used to improve chronic disease management. Regarding the form of delivery, DTx products can be delivered via mobile apps, wearable devices, virtual reality (VR) and augmented reality (AR), web-based platforms, or a combination of these technologies.

DTx products have the potential to transform modern healthcare systems (7). The clinical value includes improved patient outcomes and enhanced adherence to treatment plans. The economic benefits include reduced need for in-person visits and hospitalizations (thereby sparing the burden on healthcare systems), reduced healthcare costs for patients and providers, as well as easier scalability of the DTx solutions compared to the traditional healthcare interventions (8, 9). Furthermore, DTx solutions can improve access and convenience to medical services, which is particularly beneficial for patients in remote or underserved areas. Many DTx solutions provide highly personalized treatment interventions, accounting for the real-time monitoring of individual patient data and making dynamical adjustment of treatment plans. Finally, the data collected by DTx products in aggregation can add value to medical research, thereby improving the development of novel safe and efficacious treatments at scale.

DTx products target a wide range of disease areas, and the scope of applications will grow as the industry develops (10). The development of DTx products may be accelerated compared to that of typical pharmaceutical products. This is partly due to that DTx investigational products efficiently leverage the use of software and existing digital platforms and technologies, and because some steps in “traditional” drug development such as animal/toxicity studies are not applicable in DTx development (3, 7, 11, 12).

The present paper focuses on design and development aspects of a DTx solution for post-traumatic stress disorder (PTSD), an area of high unmet medical need in the spectrum of psychiatric disorders. Heart rate variability (HRV) biofeedback (BFB) is an increasingly promising tool for correcting autonomic dysfunction in PTSD, with potential to deliver improved clinical outcomes. Several wellness technologies that deliver at-home HRV-BFB are currently available in the consumer marketplace, e.g., HeartMath Inner Balance, EmWave2, Elite HRV, Optimal HRV, KYTO, and the Lief Smart Patch. With the exception of the Lief Smart Patch, which received FDA Class II approval for the treatment of anxiety and is thus eligible for insurance coverage in select states, the aforementioned tools are not classified as medical devices. As such, currently they cannot be officially prescribed by licensed healthcare professionals, are not eligible for insurance coverage, and generally do not include integrated mental healthcare support by licensed mental health clinicians.

The aim of this paper is to outline a holistic strategy for designing, building, testing, scaling, and distributing a prescription HRV-BFB medical device for the treatment of PTSD, which would be eligible for insurance coverage and would entail consistent mental healthcare support by licensed clinicians to greatly increase treatment access while also adhering to a rigorous standard of care. Section 2 provides background on PTSD and HRV as a therapeutic target in PTSD treatment. Section 3 outlines a strategic plan for design, calibration, clinical testing, and regulatory and commercial considerations of an HRV-BFB digital intervention. Section 4 concludes with a summary and a discussion of additional considerations for the future work.

## 2 Unmet medical need

Post-traumatic stress disorder (PTSD) is a psychiatric condition that emerges after exposure to a severely adverse event, defined in PTSD diagnostic of The Diagnostic and Statistical Manual of Mental Disorders, fifth edition (DSM-5) as follows: the adverse event must include exposure to death, threatened death, actual or threatened serious injury, or actual or threatened sexual violence. This can occur through direct personal experience, by witnessing the event in person, by learning that the event happened to a close relative or friend, or by indirect and repeated exposure to aversive details of such events, usually as a first responder (13). Other DSM-5 diagnostic criteria for PTSD include re-experiencing of the event in the form of nightmares, flashbacks, or intrusive thoughts; avoidance of situations, stimuli, thoughts, and feelings that remind the person of the event; and hypervigilance/hyper-arousal, often expressed as excessive or maladaptive threat monitoring and an exaggerated startle response. Development of PTSD following a traumatic exposure is multifactorial, and is mediated by predisposing biological factors like altered stress processing pathways (14, 15) and social factors like low socioeconomic status, female gender, and lack of social support in the aftermath of the event (16).

Estimates for the lifetime prevalence of PTSD range from 3.4% to 26.9% in the general civilian population, and range from 7.7% to 17% in the military population (17). A large epidemiological study in 2,010 (*n* = 34,653) estimated the lifetime prevalence of PTSD at 6.4% (18). Remission rates in PTSD vary widely. In a systematic review and meta-analysis of long-term outcome studies, Morina et al. (19) found an average remission rate of 44% (*n* = 81,642) within a 40-month period. A systematic review by Santiago et al. (20) found that approximately 39% of study participants (*n* = 9,570) exhibited a chronic course of PTSD. In a sample of adults who satisfied diagnostic criteria for PTSD currently or at some prior point in their lifespan (*n* = 1,997), remission was achieved by only 25.3% of women and 24.3% of men (21). Chapman et al. (22) applied advanced statistical modeling to estimate that in the general population (*n* = 8,841), 92% of participants with suspected PTSD would eventually cease to satisfy diagnostic criteria for PTSD; however, the authors calculated a median time to remission of 14 years.

### 2.1 PTSD background

PTSD causes significant functional impairment that can contribute to quality of life (QoL) outcomes like unemployment and homelessness, which are significantly more common among PTSD sufferers than in the general population (23). Additionally, approximately 80%–90% of PTSD cases present with psychiatric comorbidities (24), p. 417). Mood disorders, anxiety disorders, substance use disorders (SUD), suicidality, psychosis, and borderline personality disorder (BPD) are the most frequently assessed comorbid psychiatric conditions. Major depressive disorder (MDD) is present in more than half of PTSD cases (25) and comorbid SUD was identified in 46.4% of individuals with PTSD in a large epidemiological study (*n* = 34,653) (18). PTSD is also associated with increased risk of several significant physical morbidities, including but not limited to cardiovascular, neurological, musculoskeletal (26) and autoimmune (27) conditions.

The current standard of care in psychological treatment for PTSD emphasizes exposure-based, desensitization-oriented interventions like prolonged exposure therapy (PET), imaginal exposure (IE), trauma-focused CBT (TF-CBT), and eye movement desensitization and reprocessing (EMDR). Pharmacotherapy might be applied to target specific symptom clusters, e.g., a selective serotonin reuptake inhibitor (SSRI) for management of affective symptoms or prazosin for the reduction of hyperarousal symptoms. Ample evidence supports the favorability of exposure-based therapies over control conditions in promoting quality of life (28) and symptom reduction (29, 30) in PTSD patients. Despite the relative efficacy of exposure-based interventions, such approaches do require individuals to re-experience disturbing elements of the traumatic exposure that led to PTSD pathology. Lewis et al. (31) performed a systematic review and meta-analysis of dropout rates from RCTs of 28 distinct psychological therapies for PTSD, some of which apply exposure/desensitization methods and others that do not. The authors found “evidence that psychological therapies with a trauma focus were significantly associated with greater dropout” (p. 1). Non-trauma-focused therapies have shown moderate to large effect sizes (ibid.) and multicomponent TF-CBT has been shown to outperform “purely trauma-focused” interventions (32).

Studies of HRV-BFB for PTSD (33, 34), as well as a meta-analysis of such trials (35), have indicated high adherence to and acceptability of this non-exposure-based intervention. Given the previously discussed unmet medical need in PTSD, it is possible that a subset of patients for whom exposure-based treatments have shown limited efficacy or acceptability might be well served by psychophysiological interventions like HRV-BFB. Such interventions offer a unique opportunity to beneficially regulate the nervous system and gradually attenuate the dysfunctional stress response patterns seen in PTSD, without the need for re-exposure and related adverse effects or attrition. This regulation can have significant downstream effects on baseline physiological functioning, potentially reducing individual and population-level risk for physical pathologies that are associated with PTSD (26, 27). It is thus possible that another subset of PTSD patients—those presenting with comorbid physical pathologies driven by autonomic dysfunction—might additionally benefit from psychophysiological treatments. With these aims in mind, the health and functioning of the autonomic nervous system, as indexed by heart rate variability (HRV), is a promising target for psychophysiological intervention.

### 2.2 Autonomic pathophysiology in PTSD

The autonomic nervous system (ANS) coordinates diverse physiological processes related to survival. The sympathetic branch of the system drives allostatic and arousal processes, including fight-flight-or-fawn. The parasympathetic branch drives homeostatic and restorative–“rest-and-digest”–processes. The traumatic experiences defined in Criterion A are unified in that they produce overwhelming feelings of horror, fear, and/or helplessness that potently activate the sympathetic branch of the autonomic nervous system. The persistent re-activation of the stress response due to the hypervigilance and hyperarousal symptoms of PTSD further potentiate the sympathetic system, while also compromising parasympathetic functioning.

In PTSD, dysregulation of the stress response leads to lowered thresholds for sympathetic activation, contributing to chronic threat sensitivity that perpetuates excessive activation of these systems. Simultaneously, top-down inhibitory capacity of the prefrontal cortex (PFC) is impaired and functional connectivity between the PFC and limbic regions is compromised, contributing to emotional dysregulation. These interwoven processes reinforce a maladaptive bias toward the defensive autonomic state of sympathetic dominance. Chronic activation of the sympathetic system is metabolically taxing. Allostatic overload occurs when the demands for adaptation are too high; the stress response becomes dysfunctional, propagating inflammation, and a morbidogenic internal milieu ensues. Allostatic overload has been shown to increase risk for metabolic, cardiovascular, immunological, neurological, musculoskeletal, and psychiatric pathology (36). Indeed, substantial evidence has shown a link between PTSD and cardiovascular disease (37–41). Psychophysiological interventions have the potential to reestablish balance between homeostatic and allostatic processes, exerting systemic effects on mental and physical health outcomes.

### 2.3 Heart rate variability as a therapeutic target in PTSD treatment

Heart rate variability (HRV) refers to the variation in intervals between heart beats. HRV can be interpreted as a broad indicator of the health and functionality of the parasympathetic system. High HRV suggests that the parasympathetic system is sufficiently strong and agile to exert inhibitory force on the sympathetic system when needed, allowing the body to return to homeostasis after a detected threat has passed. When HRV is low, the parasympathetic system is less effective in restoring homeostatic function, allowing sympathetic activation to persist even once a threat has been resolved or avoided. Put another way, high HRV indicates high autonomic flexibility, wherein the autonomic nervous system is able to toggle smoothly and effectively between states of arousal and rest in accordance with changing conditions of the external environment and internal milieu. Moss and Shaffer (42) summarize: “HRV is a medical index for morbidity and wellness. Lower HRV accompanies many illnesses; high HRV accompanies healthy states, resilience, and optimal functioning” (p. 2).

Low HRV has been consistently measured in people with PTSD (43–46), and low HRV has been suggested as an indicator of autonomic dysfunction in PTSD (47, 48). Low autonomic flexibility reinforces the overgeneralized, hyperreactive threat detection and stress response pattern seen in PTSD. Importantly, this feedback loop can be reversed by targeting and enhancing autonomic function, reflected in measures of HRV. As HRV increases, the individual spends less time in sympathetic dominance, resulting in attenuation of PTSD hypervigilance/hyperarousal symptoms. Subsequently, the threshold for sympathetic activation is raised and maladaptive states of sympathetic dominance become even less frequent. Higher HRV is associated with improved emotion regulation and reductions in anxiety and rumination (49), which can further mitigate sympathetic hyper-arousal by strengthening the virtuous cycle between emotional well-being and autonomic health. Additionally, Khodik (50) demonstrated that HRV predicted self-regulatory capacity and reduced negative affectivity during experiences of acute stress in adults being treated for alcohol addiction. This is clinically relevant to the treatment of PTSD due to the 46.4% comorbidity rate with SUD noted earlier.

HRV-BFB has been suggested as a viable complementary therapy in the treatment of PTSD (51, 52). Evidence from animal models suggests that vagus nerve stimulation, a psychophysiological intervention with notable similarities to HRV-BFB, can facilitate the extinction process in conditioned fear responses (53). In some patients with PTSD, HRV-BFB might thus enhance the efficacy of exposure-based interventions. HRV-BFB has been shown to produce clinically significant benefits when applied to a variety of physical and mental illnesses. HRV-BFB interventions led to reductions in anxiety and depressive symptoms (54, 55), perceived stress (54), and panic symptoms (56). Meta-analyses conclude that HRV-BFB “is associated with a large reduction in self-reported stress and anxiety” (57), p. 2) and there is a mean medium effect size of HRV-BFB for reducing depressive symptoms (58). Research has consistently demonstrated the beneficial effect of HRV-BFB on self-regulatory processes. A systematic review of the evidence for HRV-BFB in children and adolescence found broad positive effects on physical and mental health conditions, noting that “HRV biofeedback helped to (1) improve several symptoms, (2) reduce disruptive behaviors, (3) enhance autonomic and emotional self-regulation, (4) reduce self-reported anxiety and pain levels, and (5) improve cognitive functioning” (59), p. 15). Recent fMRI and MRI evidence indicates that HRV-BFB increases cerebral blood flow (60), p. 110) and increases functional connectivity (61) between limbic and prefrontal brain regions involved in coordinated self-regulatory processes.

A systematic review and meta-analysis by Lehrer et al. (60) examined 58 RCTs of HRV-BFB covering a wide variety of clinical and physiological outcome measures. They found a significant small to moderate effect size favoring HRV-BFB across these studies. Although effect sizes for HRV-BFB did not generally exceed those of other evidence-based treatments included in the review, these comparisons included biomedical interventions like inhalers for asthma or beta blockers for hypertension. As such, even comparable efficacy of HRV-BFB as a non-pharmacological intervention is noteworthy. A systematic review by Fournié et al. (62) showed that HRV-BFB has high feasibility and no adverse effects in patients with chronic illnesses, and “significant positive effects were found in various patient profiles on hypertension and cardiovascular prognosis, inflammatory state…[and] sleep disturbances” (p. 1). In the total sample of 1,127 participants across 29 studies, no participants reported dissatisfaction with HRV-BFB and “overall, patients reported satisfaction in stress reduction and positive emotion enhancement during biofeedback and maintained long-term persistent benefits” (Ibid, p. 7). However, the authors also state that one of the reviewed studies reported slight anxiety in response to the perceived pressure of needing to meet the biofeedback protocol targets during training.

In a critical review of 14 studies investigating HRV-BFB, Wheat & Larkin (63), p. 238) note that “no studies reported any information indicating that participants perceived the treatment as adverse.” Others report that “presently there are no known contraindications or risks associated with HRV BFB, although breathing at resonance frequency for more than a few hours a day may theoretically be iatrogenic” (64), p. 7). Similarly, a review of 223 studies investigating voluntary slow breathing, which mirrors the breathing pattern applied during HRV-BFB, found minimal adverse effects like mild lightheadedness (65). Two of the studies reviewed by Fournié et al. (62) addressed this concern by incorporating a familiarization period in which participants gradually acclimated to the target breathing rate. Mayo Clinic (66) confirms the overall safety of biofeedback but suggests that those with specific medical conditions, such as heart arrhythmias or skin diseases, consult with their doctors before use. Such considerations should be accounted for during clinical trials and applications of HRV-BFB DTx.

Although high-quality, well-controlled studies of HRV-BFB in the clinical PTSD population are limited, initial evidence underscores the viability of this intervention. Bell et al. (67) conducted a controlled comparison of neurofeedback (*n* = 12) and HRV-BFB (*n* = 11). Both interventions led to very large and statistically significant reductions in PTSD symptom severity. Criswell, Sherman, and Krippner (68) used a pre/post study design without control or comparison groups and found that all adult outpatients with a PTSD diagnosis (*n* = 30) who participated in individual CBT with an HRV-BFB component fully remitted by the conclusion of the intervention. This was a modular, multimodal, symptom- and skill-specific design in which HRV-BFB was only applied during a hyperarousal and reactivity module. Patients reported satisfaction with the skills-based structure and appreciation for developing tools to apply independently in the future. The aforementioned systematic review by Fournié et al. (62) included four studies of HRV-BFB for PTSD. Results indicated improvements in sleep quality and memory, as well as reductions in depression and PTSD-specific symptoms. A meta-analysis of five studies on HRV-BFB for PTSD in military service members (*n* = 95) found a moderate to large mean effect size of HRV-BFB on PTSD symptom severity and showed a cumulative attrition rate across the five studies of only 5.8%, compared to attrition rates of 16%–36% commonly seen in existing evidence-based treatments for PTSD (35). Tan et al. (34) investigated HRV-BFB as compared to treatment as usual (TAU) for veterans with PTSD and found that the intervention “significantly increased the HRV while reducing symptoms of PTSD” and “the TAU had no significant effect on either HRV or symptom reduction” (p. 27). 95% of participants completed the study protocol and 90% reported satisfaction with the treatment and its benefits, explaining that HRV-BFB “has helped me teach myself how to control my own PTSD symptoms” (34), p. 34). Schuman et al. (33), p. 62) note that “refusal and dropout rates for PTSD treatment are particularly high among veterans due to the avoidance symptoms that characterize PTSD and the intensive trauma-focused nature of PTSD interventions” and as such, “even modest improvement from brief interventions veterans find acceptable is preferable to no treatment at all.” They also suggest that HRV-BFB may be well-suited to primary care settings, bypassing the potential stigma associated with seeking mental healthcare.

The cumulative evidence strongly suggests that HRV-BFB can be an effective and acceptable intervention that produces clinically meaningful changes in several physical and mental health outcomes, including many that are directly relevant to PTSD and associated morbidity. Given the significant unmet need in this clinical population, efforts should be undertaken to develop, test, regulate, and deploy prescription HRV-BFB using established pathways for evidence generation and digital therapeutic development.

## 3 Digital therapeutic design and development

Designing and developing a digital therapeutic (DTx) for PTSD will require an application of a strategic approach combining best methods and practices from the drug development and the software development fields (69). A systematic approach, similar to the one described by Clancy (70) could be utilized. The building elements of this approach include: (1) The Core strategy specifying the unmet medical need, the target patient population, and the intended use of a DTx solution; (2) The Build strategy specifying the product surface area defined by the form factor, mechanism of action and the technology platform of a DTx solution; (3) The Evidence Generation strategy consisting of the planned clinical trials for generating evidence base for the DTx product; (4) The Regulatory strategy outlining the pathway to obtain regulatory clearance and marketing authorization; and (5) The Commercialization strategy outlining how the DTx product could be scaled up and turned into a business.

These five strategic elements are interrelated and would have to be customized for a particular DTx candidate product. In what follows, we describe them in detail in the context of a hypothetical project to develop a novel HRV-BFB digital intervention.

### 3.1 The core strategy

Defining the target value proposition of the product is critical as it will determine the subsequent development steps and the likelihood of product success. The ideation process would involve reviewing the relevant literature on the disease of interest (e.g., PTSD), the existing treatments, and the medical gaps that can be addressed with a novel DTx solution. A clear definition of the target population is essential. One may start with a relatively narrow target population (e.g., patients who are thought to most likely benefit from the DTx solution), and potentially broaden it as the project develops. For instance, the initial focus may be on adult outpatients with the PTSD diagnosis and a history of mental health issues such as depression or anxiety.

The purpose of a novel DTx solution could be stabilization of the patient's condition during a 12-week treatment period, that would result in reducing PTSD symptoms, improving sleep, enhancing coping skills and daily functioning, etc. The DTx intervention could be designated/prototyped as a prescription digital therapeutic (PDT) consisting of a wearable device that measures heart rate and provides real-time feedback on HRV. It would be connected to a patient's smartphone and a physician's dashboard who can monitor the patient's progress and provide support and guidance as needed.

If the investigational product demonstrates promising efficacy and safety in clinical studies in a relatively small, well-defined population, later it can be scaled up to a broader population of patients with PTSD, such as veterans and military personnel, survivors of accidents and natural disasters, etc. However, the content of a DTx solution may have to be tailored to the corresponding population, considering the medical needs, patient preferences, and the treatment goals. The principles of user-centered design (UCD) (71) and personalized medicine (72) are fully applicable in this context.

### 3.2 The build strategy

One of the primary challenges in regulating HRV-BFB and other digital therapeutics is the dynamic nature of software development. There are numerous possibilities for building a DTx solution, and it is not known upfront which option would be optimal in practice. According to Clancy (70), the Build strategy can be determined by the form factor or the mode of delivery (e.g., via a smartphone app, virtual reality headset, etc.), the mechanism of action (which can vary broadly—from cognitive-behavioral therapy to interventions at the physiological level), and the technology platform that would integrate various components of the intervention into an engaging, reliable, and secure digital medicine product.

For instance, an HRV-BFB digital intervention to help individuals regulate their autonomic nervous system and improve well-being may include: (i) screening tools to perform an initial assessment of a patient to obtain their relevant medical history, baseline HRV measurements, and psychological assessments to identify any specific issues and goals; (ii) training sessions designed for an individual patient to learn how to control their HRV through biofeedback; (iii) breathing exercises designed to determine a breathing pattern for an individual to maximize HRV and help achieve a state of relaxation; (iv) regular HRV-BFB practice sessions delivered through a mobile app (prompted or on-demand); (v) monitoring tools that enable keeping track of HRV measurements, psychological and physiological outcomes, and make adjustments to the intervention, if necessary; and (vi) online support and guidance from a trained practitioner. One should be mindful of several potential challenges in the development of these design elements; for instance, ensuring that data collection platforms are robust and reliable, individual patient data are properly encrypted and protected, the embedded assessments reflect well-defined, validated outcome measures (73), the prompted protocols are easy to follow to maximize patient engagement and adherence (74), and prompt medical and technical support is available (75).

Technological platforms for digital therapeutics in PTSD treatment are evolving to offer innovative features aimed at enhancing monitoring and intervention strategies. Passive 24-h monitoring through wearable devices combined with prompted interventions represents a powerful approach to continuous patient support (76). Physiological and behavioral metrics can be collected around the clock, providing valuable insights into the patient's daily experiences and potential triggers for PTSD symptom exacerbation (77). Prompted interventions, triggered by predefined thresholds, enable timely symptom mitigation. For instance, if a wearable device detects a sudden decrease in HRV or a sleep disturbance indicative of distress, the platform can prompt the patient to engage in relaxation techniques or connect with an integrated support resource (78, 79). This real-time support can help individuals manage symptoms more effectively and prevent escalation. Such devices and applications could be synchronized with electronic health records (EHRs) to allow healthcare providers to monitor patient progress and make data-driven adjustments to treatment plans. This integration ensures a personalized and dynamic approach to PTSD treatment, leveraging the benefits of both digital and conventional methods.

Additionally, the integration of individualized “tag” banks facilitates more granular data tracking, allowing patients to annotate specific events or experiences associated with their PTSD symptoms. Studies exploring the use of ecological momentary assessment (EMA) in mental health research provide a foundation for the use of tag banks. EMA involves collecting real-time data on individuals' experiences, behaviors, and symptoms in their natural environments (80). Tags can be customized to capture diverse contextual factors, such as triggers, emotions, activities, and substance/medication use. Studies have shown that individuals with chronic illnesses or mental health conditions can effectively use mobile apps to track symptoms, treatment adherence, and contextual factors influencing their health outcomes (81).

The integration of screening, monitoring, and prompted intervention tools could offer a general multifaceted approach to PTSD management; as such, it would constitute a “complex intervention” (82) that would have to be tailored to an individual user through interactive testing and calibration.

### 3.3 The evidence generation strategy

Once a prototype DTx solution has been developed, it will undergo testing in clinical trials that will form the evidence base for subsequent submission and marketing authorization. Espie & Henry (83) provide an excellent overview of strategic aspects for developing a clinical research program for a DTx, the types of clinical study designs, and levels of evidence. Similar to drug development, there is no “one size fits all” strategy for evidence generation of a DTx. However, in contrast to drug development, a DTx development program will be more agile, resembling software development. With a DTx investigational product, some studies that are mandatory for a pharmaceutical product development (e.g., animal toxicity studies) are not applicable, and therefore DTx development timelines will be streamlined compared to drug/biologic development.

In the realm of DTx for PTSD, evidence-generation strategies align more closely with software development paradigms than traditional drug development methods (75). The iterative nature of software development is mirrored in the agile development processes employed by DTx companies. Rather than following the linear and time-intensive phases of traditional drug development, which include preclinical research, clinical trials, and regulatory approval, DTx companies often adopt agile methodologies that allow for rapid prototyping, testing, and refinement of their products based on user feedback and real-world data (84).

In general, the evidence generation strategy will be primarily driven by the target value proposition, regulatory and commercial considerations (70). The early development of an HRV-BFB intervention will include studies to assess usability, accessibility, and feasibility of the solution. For instance, a usability/feasibility study will focus on user experience, ease of use, and overall satisfaction from both patients with PTSD and professionals (healthcare providers) who would be using the intervention in a clinical setting. The design of such a study could be a mixed-method approach (85) combining surveys, questionnaires, interviews with focus groups, to help identify any usability issues and areas for improvement.

A feasibility study would typically aim at assessing whether an HRV-BFB intervention is practical, can be successfully implemented in practice, and provides preliminary evidence of efficacy. Such a study would track changes in user experience over time (e.g., before using the intervention, halfway through, and at the end of the study period). The findings would be compiled to inform the future development and implementation of the HRV-BFB intervention, and it would provide estimates of important parameters, such as recruitment rate, withdrawal/dropout rates, compliance, etc., to subsequently design a randomized controlled trial (RCT).

An important step in the development of an HRV-BFB digital solution is the calibration of intervention. To deliver maximum benefit, the intervention should be tailored to the user and the context of use. For example, the control of HRV through biofeedback may depend on individual patterns of engagement and the other characteristics of a PTSD patient. The optimal frequency of prompts to engage in the breathing exercises can vary across individuals; for some, only one notification may be sufficient, whereas for others more frequent prompts may be required. Likewise, the optimal duration of the breathing exercises can vary not only between individuals but also within the same individual. The HRV-BFB digital solution is an example of a “complex intervention” (82) whose components are subject to calibration. For calibration studies, several research methodologies can be useful, including single-case experimental designs (86), factorial designs (87), sequential multiple assignment randomized trials (88), micro-randomized trials (89), amongst others. The ultimate goal of calibration studies is to build a robust version of a digital intervention that can be tested in an RCT.

The RCT is a hallmark research design in the biomedical field (90). When properly designed and implemented, an RCT allows to obtain unbiased causal estimates of intervention effect. The essential considerations for the RCT design include randomization, blinding, and the use of a control group. A standard RCT design in 1:1 randomized, double-blind, placebo-controlled trial. The 1:1 randomization means that half of the enrolled study participants are randomly assigned to receive the HRV-BFB intervention, and the other half are assigned to the control group. The trial recruitment may be web-based, involving several study centers, and the randomization procedure may have to be implemented using Interactive Response Technology (IRT). *Blinding* in RCT refers to a process of keeping treatment assignments unknown or not easily ascertained by those involved in the conduct and interpretation of the clinical trial. Ideally, one would ensure that all of the study participants, investigators, outcome assessors, and data analysts are blinded to treatment assignment; however, in trials of digital interventions this may be challenging to achieve. *The use of a control group* is important to facilitate a proper assessment and contrast of the intervention effect. While the general principles for choosing a control group is available (91), in practice it will be context specific. For an HRV-BFB digital intervention, some possibilities include the placebo control (e.g., participants use the app that appears similar but does not give real-time feedback on HRV); the waitlist control (e.g., participants are placed on a waitlist to receive HRV-BFB after the trial period); the standard of care control (e.g., the current treatment typically provided to PTSD patients), etc.

Some additional considerations for the RCT include the choice of the sample size, the specification of inclusion and exclusion criteria to select eligible participants for the study, the choice of the primary outcome to address the clinical research hypothesis, the data analysis strategy, etc. Inclusion and exclusion criteria should account for aforementioned potential risks—however minimal—of interactions with pre-existing heart arrythmias and skin diseases (66) that might be exacerbated by electrical activity or device adhesives, respectively, or of mild discomfort associated with voluntary slow breathing practices (65) such as those employed in HRV-BFB (64). Care should also be taken to ensure that inclusion and exclusion criteria across trials generate a diverse enough sample population for results to be effectively generalizable.

The primary outcome measures for an HRV-BFB RCT could be changes in heart rate variability, stress levels, or other physiological markers. Secondary outcomes might include improvements in mental health, quality of life, or other patient reported outcomes. In addition, careful monitoring of safety should be in place. Some theoretical safety risks include potential increased anxiety or distress when engaging with digital content that triggers traumatic memories (75), worsening of symptoms or new psychological issues (92), potential discomfort during slow breathing patterns, etc.

The sample size would be chosen to have ≥80% power to detect the between-group difference (when it exists) on the chosen primary outcome using a pre-specified significance level (e.g., 5%). Importantly, recruitment and retention of a sufficient sample represent significant challenges in the RCT process; these issues are potentially heightened in trials focused on the PTSD patient population (93–95). Throughout the trial, individual participant data would be acquired at multiple time points to assess treatment effect over time. The statistical analysis strategy for a continuous outcome such as HRV measured repeatedly during the course of the trial may include a mixed effects model with repeated measurements (96) with treatment contrast estimated at the end of the treatment period as the primary comparison. Patient engagement may be an important mediator of the efficacy of HRV-BFB; therefore, “exposure-response” analysis may provide additional insights into how efficacy changes for different degrees of individual patient engagement (97). Ideally, the results of an RCT would be published in a peer-reviewed scientific journal, providing evidence for the effectiveness (or lack thereof) of the intervention.

It is important to note that there may be numerous approaches for developing the clinical evidence base. Depending on strategic goals, several RCTs may be conducted, in the same or different patient populations, with possibly different control groups. *Adaptive designs* that allow pre-specified modification of one or more aspects of the trial based on accumulating data while maintaining the validity and integrity of study results can be considered (98). For instance, an adaptive enrichment design to assess the efficacy of HRV-BFB can start with a broad population of PTSD patients, and then based on the results of an interim analysis may shift the focus to a subpopulation of patients for whom there is emerging evidence of a very strong effect of the HRV-BFB intervention. *Master protocols*, such as basket, umbrella, and platform trials (99) may be also useful in this context. For instance, the sponsor may designate a master protocol for one broad indication (PTSD) and have several sub-studies evaluating different versions of the HRV-BFB intervention tailored to a particular patient subgroup (e.g., veterans, military, survivors of accidents and natural disasters, etc.). Multi-arm trials, where different arms receive integrated care combining DTx with other interventions, such as pharmacotherapy, allow for the assessment of synergistic effects.

Throughout the trial process, safety of the interventions remains paramount, requiring continuous monitoring and risk-benefit evaluation. Unlike traditional drug development, where adverse events primarily relate to chemical compounds, DTx safety considerations may encompass issues such as data privacy and cybersecurity. Regulatory strategies intersect with build strategies and evidence generation strategies to incorporate such considerations into product development.

### 3.4 The regulatory strategy

The regulatory strategy will be driven by the target value proposition of the product, and it will be also influenced by the commercial considerations. Consultations with health authorities will be very important to ensure compliance with the regulatory requirements. In the US, the Food and Drug Administration (FDA) Center for Devices and Radiological Health (CDRH) is primarily responsible for the regulatory oversight of medical devices and digital therapeutic interventions. In general, the regulation process follows a risk-based approach for which devices are classified as Class I (lowest risk of harm to patients or users), Class II (intermediate level of risk), or Class III (potential unreasonable risk of illness or injury). Class I-II products may be exempt from premarket clearance or approval requirements, whereas Class III products would be required to obtain Premarket Approval (PMA). HRV-BFB products intended for general wellness, such as stress reduction or relaxation, may be considered low-risk and might be exempt from premarket review under the FDA's general wellness policy. However, when HRV-BFB claims to diagnose, treat, or prevent specific medical conditions, it is classified as a medical device and subject to more stringent regulatory requirements. The two DTx that are most directly relevant to the current proposal are the Lief Smart Patch, an HRV-BFB device approved for the indication of anxiety, and Freespira, a device that retrains CO2 hypersensitivity and is approved for the indications of PTSD and panic disorder. Notably, both of these devices received Class II designation by the FDA.

To determine a proper regulatory pathway for an HRV-BFB digital intervention, early engagement of the sponsor with the regulators would be essential. For instance, the agency may categorize the intervention as Class II or III, which could potentially be developed following a *de novo* classification process (100), or the Pre-Cert program (101). Unlike traditional medical devices, digital therapeutics often undergo frequent updates and iterations. This rapid development cycle poses difficulties for regulators who must ensure that each version maintains compliance with safety and efficacy standards. The FDA's Software Pre-Cert Program is an attempt to address this challenge by shifting the focus from premarket review of individual products to the evaluation of the software developer's culture of quality and organizational excellence (102).

From the sponsor side, the regulatory strategy would be planned taking into consideration the following components: (i) pre-submission activities, including meetings to discuss the regulatory pathway requirements and concerns related to the HRV-BFB digital intervention; (ii) clinical development plan (cf. “Evidence Generation Strategy”); (iii) regulatory submission package, including the necessary information on HRV-BFB, its intended use, and the relevant experimental evidence; (iv) clinical trial data and reports, including detailed statistical analyses to demonstrate the safety and efficacy of the HRV-BFB digital intervention; (v) marketing authorization application; and (vi) post-marketing surveillance plan to monitor the safety and effectiveness of the intervention in real-world use.

If the target market is global (e.g., US, Canada, Europe, Asia, Australia, etc.), some additional regulatory considerations may apply. The regulatory landscape for HRV-BFB varies significantly across regions, reflecting differences in regulatory philosophies and healthcare systems. In the European Union, the regulatory framework is governed by the Medical Device Regulation (MDR), which came into full effect in May 2021 (103–105). The MDR has strengthened the requirements for clinical evaluation and post-market surveillance for medical devices, including digital therapeutics. HRV-BFB products classified as medical devices must undergo a conformity assessment process, which involves a notified body that evaluates the product's safety and performance based on clinical evidence (106). The classification of HRV-BFB devices within the MDR framework depends on their intended purpose and risk profile, with higher-risk devices requiring more rigorous assessment.

### 3.5 The commercialization strategy

The key considerations for building the commercialization strategy include the *Customer* decision, the *Distribution* decision, and the *Payment* decision (70).

To select the right customer, market research and analyses would have to be conducted early on, to understand the target population of patients with PTSD, their needs and pain points, as well as analyzing the competitors' offerings. The number of target customers, their willingness to pay, the evidence requirements for the digital therapeutic solution, and the projected time scales for relevant sales cycles will inform the subsequent steps in product development, testing, validation, and regulatory submission.

The distribution decision is dependent on the chosen customer. There are several models for the distribution of a DTx product, such as direct-to-consumer, payer-to-patient, and provider-to-patient. A marketing strategy would include the development of a strong brand and positioning of the HRV-BFB digital intervention such that it resonates with the target customer. A robust sales and distribution network would need to be in place to ensure the product is easily accessible to the customer; this may require partnerships with healthcare providers, online retailers, and brick-and-mortar stores. In addition, customer support and engagement (e.g., technical support, educational resources, engagement with users through various channels to gather their feedback) would be important for the successful marketing of the product.

Finally, for the payment decision, it is important to consider options that are convenient and accessible for the chosen customer. For example, if the HRV-BFB intervention is delivered through a mobile app, in-app purchases can be a convenient way to pay for additional features or premium content. Offering a subscription service where the users are billed monthly, quarterly, or annually for access to the intervention may be a useful option. If the HRV-BFB intervention is recognized as a prescription digital therapeutic (PDT), it may be possible to work with insurance companies to offer reimbursement for users.

## 4 Discussion

### 4.1 Summary of proposal

Abundant evidence outlines a significant unmet clinical need regarding PTSD disease burden, widespread autonomic pathophysiology associated with this condition, and the potential therapeutic efficacy of HRV-BFB for enhancing autonomic function with corresponding improvements in key clinical and patient-reported PTSD outcomes. HRV-BFB can facilitate self-regulatory and psychophysiological processes that may support partial or full remission of PTSD symptoms, while also reducing the risk of common physical and mental comorbidities and functional impairments that further compromise QoL in people with PTSD.

Existing and emerging pathways for developing, testing, regulating, and distributing DTx should be utilized to further investigate the efficacy of HRV-BFB for supporting and enhancing patient outcomes in PTSD treatment. Thoughtful development and scaled distribution of a viable HRV-BFB intervention for PTSD could produce significant improvements in individual and population-level outcomes, given that the current treatment landscape fails to achieve timely remission for the majority of PTSD patients. Careful consideration should be given to factors affecting availability and accessibility of prescription HRV-BFB for PTSD, as well as factors affecting patient engagement–many of which may be specific to the clinical features of PTSD and/or individual variables that mediate course and prognosis in this condition.

This paper represents a novel proposal for the development, evaluation, and deployment of a prescription HRV-BFB DTx in PTSD treatment, based on a robust evidence base supporting the potential viability of this intervention to help reduce the unmet medical need in this clinical population. The proposal offers a high-level holistic framework upon which researchers and DTx developers can build specific approaches within and across each of the five strategies outlined in Section 3. One limitation of our proposal is that it is quite general and may be lacking some important recommendations for clinical development teams and other relevant stakeholders at the tactical level. This was done intentionally as the nuances of patient demographics, current standards of care and access to them might suggest varying intervention components for maximizing the outcome. We intend to address the aforementioned limitation in future work.

### 4.2 Additional considerations

Clinical trials will need to be designed with careful consideration of the defined inclusion and exclusion criteria to ensure appropriate patient selection. This may involve advocacy for the inclusion of patients with complex comorbidities commonly seen in PTSD populations (107), who may be excluded from more conventional clinical trials due to these complicating factors and concerns about elevated risk for adverse reactions. As such, special attention must be paid to mitigating the risks that are unique to individual participants and to the PTSD patient population in general. Additionally, establishing a clinical trial network with a careful triage and referral process can facilitate patient recruitment and ensure diverse representation across different settings and populations (2, 107).

Furthermore, the regulatory landscape for digital therapeutics is still evolving. Ensuring that digital health tools meet stringent safety, efficacy, and privacy standards is essential, but navigating this regulatory environment can be daunting for developers and healthcare providers alike (108). Moreover, the collaboration between healthcare providers, technology developers, regulatory bodies and insurance companies is crucial for the successful integration of HRV-BFB. This involves adhering to regulatory standards and ensuring the digital therapeutic tools are validated through rigorous clinical testing. Establishing interoperability standards, and seamless sharing of data between digital platforms and EHRs can facilitate the providence of a unified patient profile that supports comprehensive care.

Efforts towards global harmonization of digital therapeutics regulations are ongoing, with organizations such as the International Medical Device Regulators Forum (IMDRF) playing a pivotal role. The IMDRF's work on Software as a Medical Device (SaMD) guidelines aims to create a common framework that can be adopted across different jurisdictions, facilitating international market access for HRV-BFB products (109).

The regulatory landscape is likely to further evolve with advancements in artificial intelligence (AI) and machine learning (ML) (110). These technologies are increasingly being integrated into HRV-BFB solutions to enhance personalized feedback and predictive analytics. Regulators are beginning to develop frameworks for AI/ML-based medical devices, focusing on aspects like transparency, algorithmic fairness, and continuous learning systems. The FDA's proposed regulatory framework for AI/ML-based SaMD, for example, outlines a total product lifecycle approach, emphasizing the importance of real-world performance monitoring and iterative improvements (111).

Data privacy and cybersecurity are paramount concerns in the regulation of HRV-BFB. These products collect and process sensitive health data, necessitating stringent measures to protect user information from breaches and unauthorized access. Regulatory bodies like the FDA and the European Data Protection Board (EDPB) under the General Data Protection Regulation (GDPR) provide guidelines and requirements for data security and privacy (112, 113). Compliance with these regulations is crucial for maintaining user trust and avoiding legal penalties.

Regarding the integration of HRV-BFB into the PTSD treatment paradigm, one challenge is inconsistent patient adherence to digital interventions. Despite the convenience of mHealth applications, maintaining regular use requires sustained motivation and engagement, which can be challenging for individuals struggling with PTSD symptoms (114). The ideation phase, which includes the *core strategy* and the *build strategy*, should account for relevant engagement barriers in the DTx design. The efficacy of such design considerations is then tested in the evidence generation strategy and design elements are refined in iterative loops, based on the results of preliminary and mid-point analyses.

Researchers and clinicians should also consider the social effects of promoting HRV-BFB as a treatment for PTSD. Through health education and promotion efforts that explicitly describe autonomic processes in PTSD, using accessible and non-medicalized language, deeply entrenched societal stigma regarding “mental” illness might be modified. By emphasizing the physiological drivers of the disorder, patients and as-yet-undiagnosed or at-risk individuals might experience less internalized stigma, which could potentially improve treatment-seeking and treatment adherence. Additionally, if RCT results show sufficiently positive advantages of HRV-BFB over existing treatments, which are known to have relatively limited efficacy and to require unpleasant re-experiencing of the initiating exposure, people with chronic PTSD may feel reasonably optimistic about the possibility of remission. This might further improve treatment-seeking and adherence.

Lastly, developers are encouraged to seek avenues for insurance coverage and reimbursement wherever possible. PTSD is associated with significant functional impairment that unfortunately corresponds with low health capital for many sufferers. Availability and accessibility of the DTx, including economic accessibility, must be primary considerations if we are to achieve meaningful reduction of the PTSD disease burden.

## 5 Conclusion

Rigorous development and effective clinical implementation of an HRV-BFB prescription DTx for the treatment of PTSD has the potential to significantly improve several key outcomes for this underserved patient population. Development strategies should build on existing evidence-based approaches and use defined regulatory pathways to maximize efficacy and produce a reliable, scalable product. This process will involve a variety of stakeholders, including but not limited to patients and patient advocacy groups, clinicians, software developers, researchers, regulatory officials, marketers, and healthcare organizations.

## Data Availability

The original contributions presented in the study are included in the article/Supplementary Material, further inquiries can be directed to the corresponding author.
